# Site of asteroid impact changed the history of life on Earth: the low probability of mass extinction

**DOI:** 10.1038/s41598-017-14199-x

**Published:** 2017-11-09

**Authors:** Kunio Kaiho, Naga Oshima

**Affiliations:** 1 Department of Earth Science, Graduate School of Science, Tohoku University, Sendai, 980–8578 Japan; 2 Meteorological Research Institute, Tsukuba, 305-0052 Japan

## Abstract

Sixty-six million years ago, an asteroid approximately 9 km in diameter hit the hydrocarbon- and sulfur-rich sedimentary rocks in what is now Mexico. Recent studies have shown that this impact at the Yucatan Peninsula heated the hydrocarbon and sulfur in these rocks, forming stratospheric soot and sulfate aerosols and causing extreme global cooling and drought. These events triggered a mass extinction, including dinosaurs, and led to the subsequent macroevolution of mammals. The amount of hydrocarbon and sulfur in rocks varies widely, depending on location, which suggests that cooling and extinction levels were dependent on impact site. Here we show that the probability of significant global cooling, mass extinction, and the subsequent appearance of mammals was quite low after an asteroid impact on the Earth’s surface. This significant event could have occurred if the asteroid hit the hydrocarbon-rich areas occupying approximately 13% of the Earth’s surface. The site of asteroid impact, therefore, changed the history of life on Earth.

## Introduction

Sixty-six million years ago, the Chicxulub asteroid impact in what is now Mexico led to ecosystem collapse including devastation of land vegetation^1^, the extinction of dinosaurs and >75% of all land and sea animals, and the subsequent macroevolution of mammals^1–5^. The coincidence of a mass extinction at the Cretaceous/Paleogene (K–Pg) boundary and the iridium (sourced from the asteroid) layer of the Chicxulub impact were demonstrated using marine microfossils and fossil pollen^6–8^. All available evidence suggests that the Chicxulub impact was the driver of the extinction.

Blocking of sunlight by dust and sulfate aerosols ejected from the rocks at the site of the impact (impact target rocks) was proposed as a mechanism to explain how the physical processes of the impact drove the extinction^2,3,9–11^; these effects are short-lived and therefore could not have driven the extinction^11,12^. However, small fractions of stratospheric sulfate (SO_4_) aerosols were also produced^12^, which may have contributed to the cooling of the Earth’s surface. The other possible cause is stratospheric soot aerosols^1,13^. Soot was recorded globally at the K–Pg boundary^1,14–17^. The source of the soot was thought to be wildfires^14,15^ and impact target rocks^1,13,17^. Soot spreading into the stratosphere leads to global cooling^1^. Stratospheric soot is not formed by wildfires, but by the burning and ejection of impact target rocks^1,13^. The ratio of soot components at the K–Pg boundary indicates a higher energy than forest fires, and the equivalence of molecular ratios at proximal and distal sites indicate that the soot was sourced from the target rocks of the Chicxulub asteroid impact^1^.

Kaiho *et al*.^1^ provided direct evidence for stratospheric hydrocarbon soot at the boundary and modeled how this would affect climate. They demonstrated that burning of hydrocarbons (mainly kerogen with smaller amounts of oil, although Kaiho *et al*.^1^ emphasized oil) in the target rocks by the asteroid impact produced massive amounts of soot^1^. The soot spread globally and efficiently absorbed and scattered sunlight in the stratosphere. They calculated global climate change using the amount of stratospheric soot and showed that the soot aerosols led to sufficiently colder climates at mid- to high latitudes and to drought with milder cooling at low latitudes on land, in addition to limited cessation of photosynthesis in global oceans from within a few months to 2 years after the impact. This was followed by surface-water cooling in global oceans within a few years^1^. Cooling, coinciding with mass extinction, was actually detected recently^18,19^. Stratospheric soot levels of approximately 350 Tg, corresponding to 150 times the volume of a baseball arena covered by a full roof, may have led to the extinction of the dinosaurs and warm water-dwelling ammonites, whereas crocodiles and cool-water-dwelling ammonites survived^1^. Mass extinctions occur when 1500 Tg of black carbon (BC, equivalent to soot) are ejected, corresponding to 350 Tg BC in the stratosphere, 8–10 °C cooling in global mean surface air temperature, and 10–16 °C cooling in global mean surface air temperature on land^1^. BC ejection of 1500 Tg from an impact would be a sufficient threshold to cause mass extinction.

The amount of hydrocarbon and sulfur in rocks varies widely depending on location. This means that a K–Pg mass extinction was dependent on the impact site. Here we calculate amounts of stratospheric soot and sulfate formed by a virtual asteroid impact at various global locations, based on their content in target rocks, and the resulting surface air temperature anomaly dependent on the impact site to demonstrate the low probability of mass extinction and the subsequent macroevolution of mammals when the asteroid impacted Earth.

## Results

### Climate changes due to varying amounts of soot

**Figure 1:**
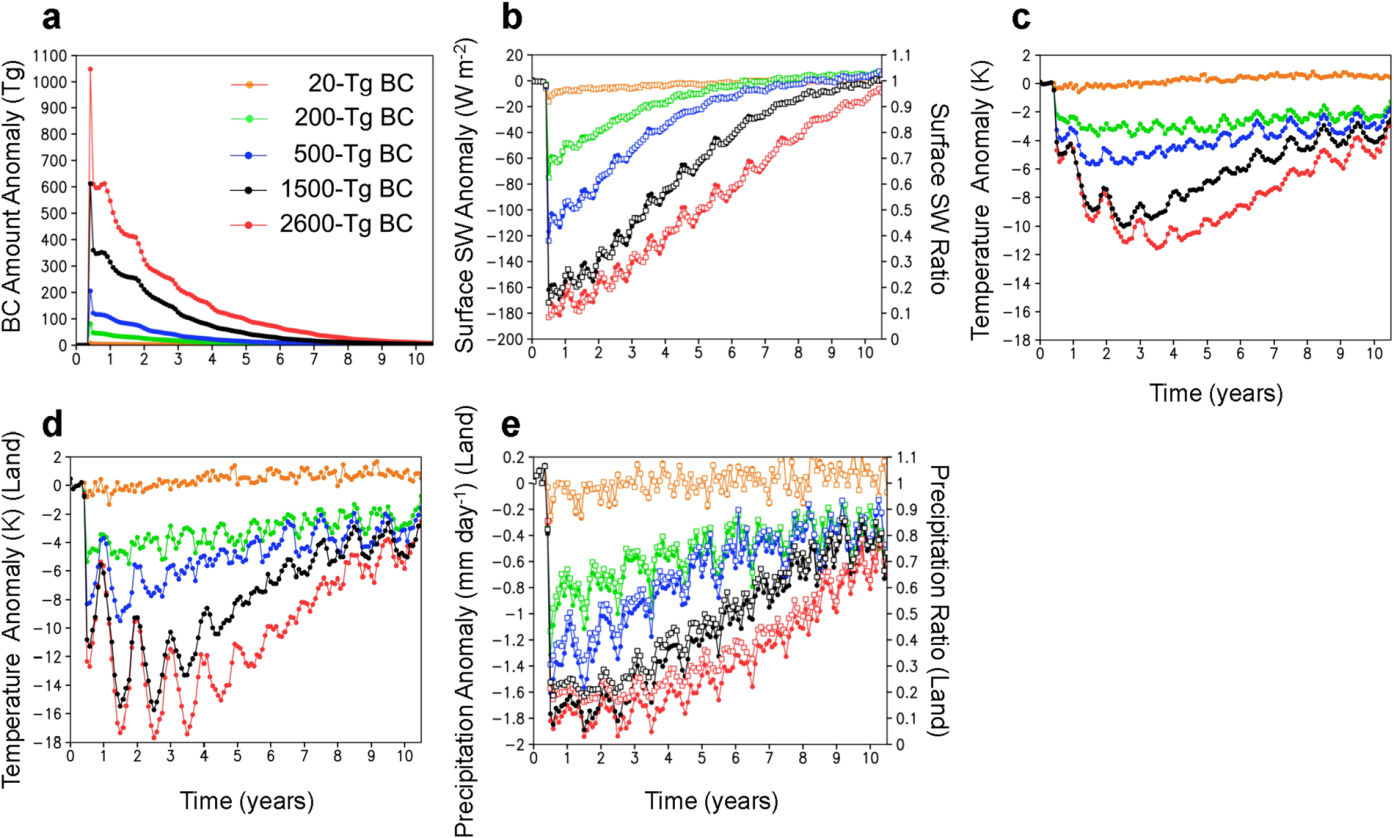
Climate changes caused by black carbon (BC) injection from ejected Chicxulub rocks. (**a**) Changes in the global averages of BC amounts in the atmosphere, (**b**) downward shortwave (SW) radiation at the surface, (**c**) surface air temperature, (**d**) surface air temperature on land, and (**e**) precipitation on land for the 20-Tg (orange), 200-Tg (green), 500-Tg (blue), 1500-Tg (black), and 2600-Tg (red) BC scenarios calculated by the climate model. Monthly anomalies from the control experiment (no ejection case) are indicated on the left axis by filled circles (**a**–**e**), and ratios relative to the control experiment are indicated for shortwave radiation and precipitation on the right axis by open squares (**b**,**e**). The 30-year global averages of the amount of BC, downward shortwave radiation at the surface, surface air temperature, surface air temperature on land, and precipitation on land in the control experiment were 41 Gg, 200 W m^−2^, 287 K, 281 K, and 2.2 mm day^−1^, respectively.

**Figure 2:**
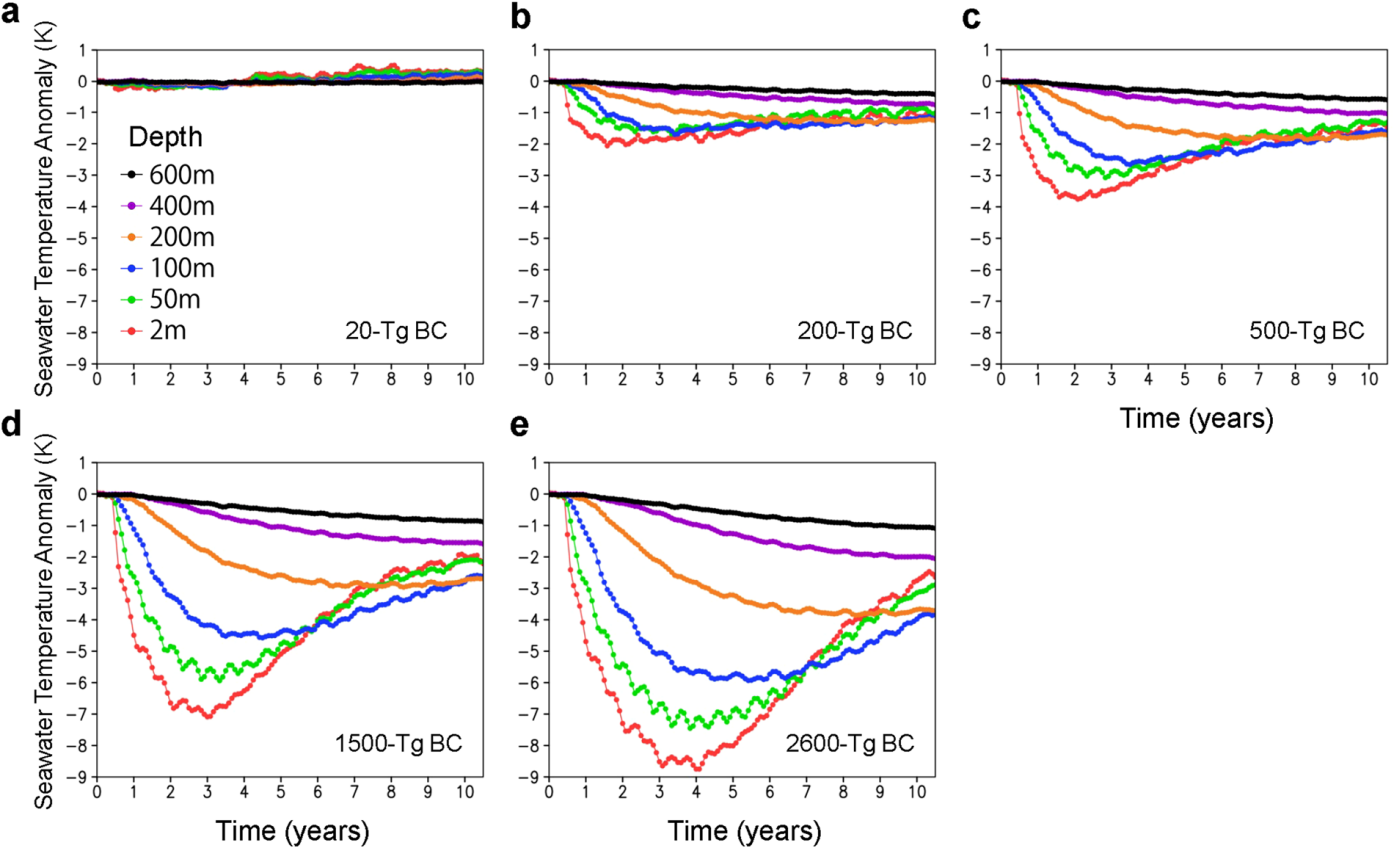
Seawater temperature changes caused by black carbon (BC) injection from ejected Chicxulub rocks. (**a**–**e**) Changes in the global averages of seawater temperature at water depths of 2 m, 50 m, 100 m, 200 m, 400 m, and 600 m for the 20-Tg (**a**), 200-Tg (**b**), 500-Tg (**c**), 1500-Tg (**d**), and 2600-Tg (**e**) BC scenarios calculated by the climate model. Monthly anomalies from the control experiment (no ejection scenario) are shown. The 30-year global averages of seawater temperature at water depths of 2 m, 50 m, 100 m, 200 m, 400 m, and 600 m in the control experiment were 293, 292, 290, 287, 283, and 280 K, respectively. The regions where seawater temperatures were below 0 °C at a 2-m water depth in the control experiment were excluded for the estimation of the anomalies and 30-year averages, to exclude the sea ice area.

Following Kaiho *et al*.^1^, we estimated climate changes caused by BC injection due to the Chicxulub asteroid impact for five quantities of BC (20, 200, 500, 1500, and 2600-Tg BC ejection cases) using global climate model calculations (see Methods). Although BC in the troposphere was efficiently removed from the atmosphere by precipitation (within approximately 1 week)^20,21^, BC in the stratosphere had a longer lifetime and was gradually deposited on the surface, on a scale of several years (Fig. 1). The stratospheric BC rapidly reduced the sunlight reaching the Earth’s surface, which led to cooling of the tropospheric atmosphere and ocean, and a decrease in precipitation on a global scale (Figs 1 and 2). These climate changes were greater for larger BC ejections. Changes in the atmosphere showed a rapid response immediately after the impact: up to 0–0.5 °C, 2–3 °C, 4–6 °C, 8–10 °C, and 8–11 °C cooling of the global mean surface air temperature, 0–1 °C, 4–5 °C, 6–9 °C, 10–16 °C, and 10–18 °C cooling of the global mean surface air temperature on land, and 0–15%, 25–50%, 45–70%, 65–80%, and 75–85% decreases in global mean precipitation on land for 20-, 200-, 500-, 1500-, and 2600-Tg BC ejection cases, respectively, within a few years after the impact; temperature and precipitation gradually recovered within the following 10 years (Fig. 1). Seawater temperature changes exhibited a slower response following the impact, and cooling at shallower water depths (<100 m) was faster and greater than cooling at greater water depths (e.g., up to 0.5 °C, 2 °C, 4 °C, 7 °C, and 9 °C decrease in global mean seawater temperature at a 2-m water depth for 20-, 200-, 500-, 1500-, and 2600-Tg BC ejection cases, respectively, within 1–4 years after the impact, and within 1 °C cooling at a 600-m water depth for all cases within >10 years).

### Climate changes due to impact latitude

**Figure 3:**
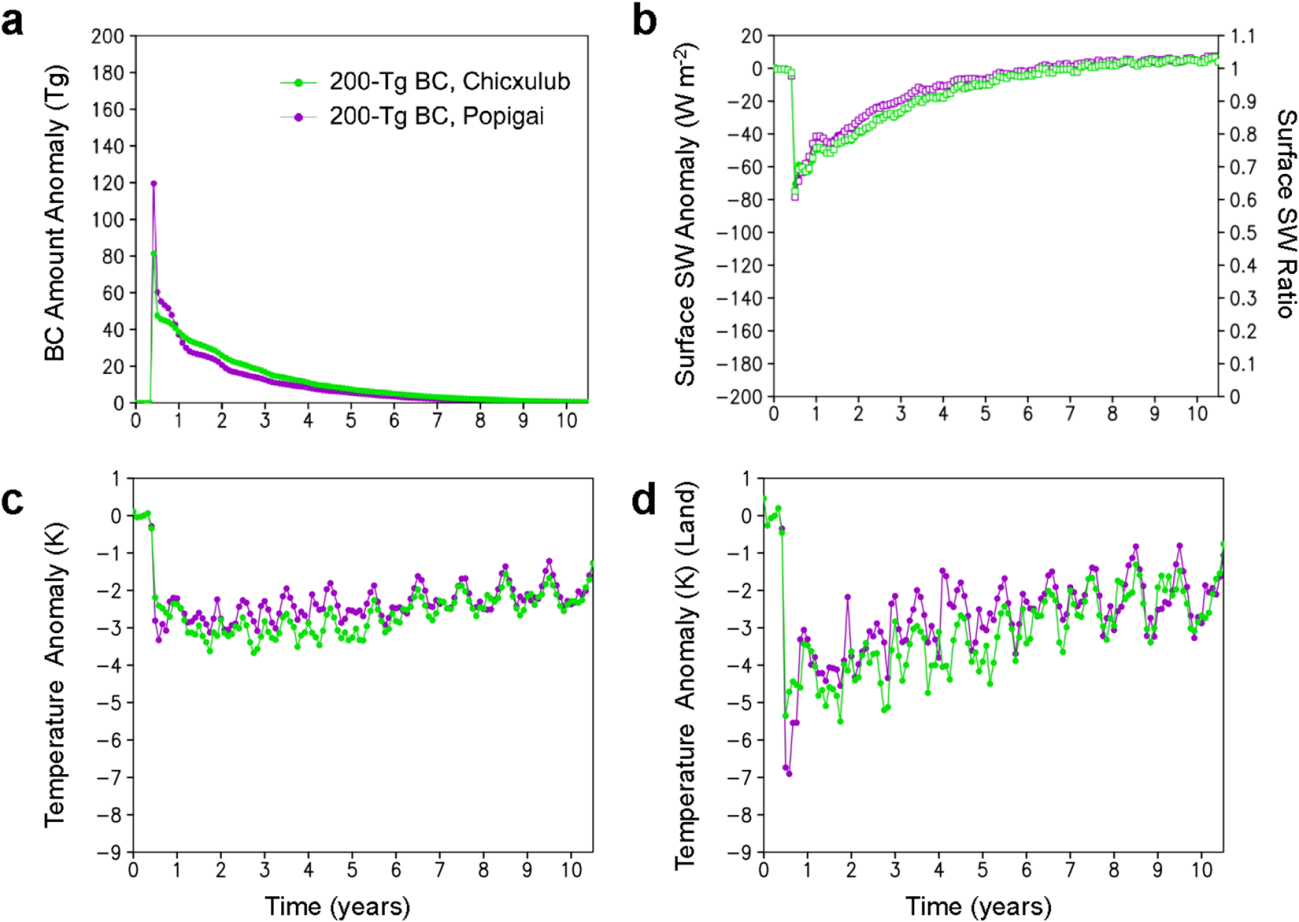
Comparison of climate changes caused by black carbon (BC) injection from low-latitude Chicxulub and high-latitude Popigai rocks for the 200-Tg BC cases. (**a**) Changes in the global averages of the amount of BC in the atmosphere, (**b**) downward shortwave (SW) radiation at the surface, (**c**) surface air temperature, and (**d**) surface air temperature on land for the low-latitude Chicxulub 200-Tg (green) and the high-latitude Popigai 200-Tg (magenta) BC scenarios calculated by the climate model. Monthly anomalies from the control experiment (no ejection case) are indicated on the left axis by filled circles (**a**–**d**), and the ratios relative to the control experiment are indicated for shortwave radiation on the right axis by open squares (**b**). The 30-year global averages of the amount of BC, downward shortwave radiation at the surface, surface air temperature, and surface air temperature on land in the control experiment were 41 Gg, 200 W m^−2^, 287 K, and 281 K, respectively.

We estimated the dependence of surface air temperature changes on location (i.e., latitude) of BC ejection for the 200-Tg BC case (see Methods). Because tropopause height is lower at higher latitudes, more BC was injected into the stratosphere in the high-latitude Popigai case than in the low-latitude Chicxulub case, leading to more cooling (approximately 1 °C both globally and on land) in the Popigai case during the first half-year following the impact (Fig. 3). However, the stratospheric BC particles ejected at high latitudes were more affected by descent during the extratropical northern hemisphere winter, and the quantity of BC in the atmosphere became similar for both cases, leading to similar cooling effects (within 1 °C on average) in the following years. The first-year averages of stratospheric soot amounts and their effect on global mean surface air temperature through impacts at low and high latitudes were similar, and differences in BC and global mean surface air temperatures were only 10 Tg and 0.5 °C, respectively (Fig. 3). We did not consider the effects of impact site latitude differences on climate changes in each case, because the differences would be minute compared to the approximately 8–10 °C global cooling, which caused the mass extinction.

### The amount of stratospheric soot and sulfate

**Figure 4:**
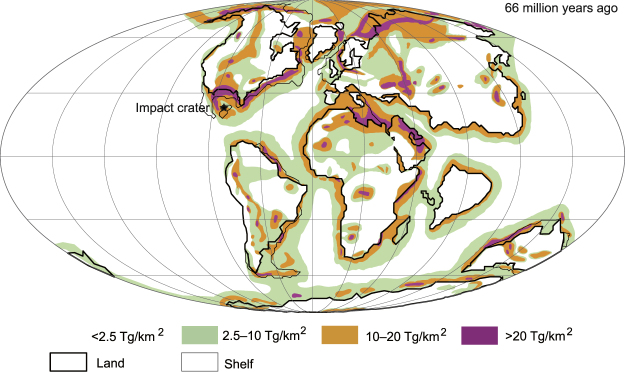
Global map showing the amount of organic matter in sedimentary rocks ejected if the Chicxulub asteroid hit various locations at the end of the Cretaceous. Shaded areas denote the following burned organic carbon weights in each area burned by the asteroid impact: white: <22,000 Tg; olive: 22,000–89,000 Tg; orange: 89,000–220,000 Tg; and magenta: 220,000–890,000 Tg. These areas correspond to 0–4 °C, 4–8 °C, 8–11 °C, and ≥11 °C cooling (global mean surface air temperature anomalies) and 0–6 °C, 6–13 °C, 13–17 °C, and ≥17 °C cooling on land by soot only, respectively, when the asteroid hit each area (Table 3). Mass extinction could have been caused by 8–11 °C or more cooling^1^ when the asteroid hit an orange or magenta area, which occupied approximately 13% of the Earth’s surface. The map is based on Courtillot *et al*.^53^; thin lines indicate continental crust shelf edges.

Tables 1 and 2 list the amounts of hydrocarbons in sedimentary rocks and sulfur in sedimentary and mantle rock in various locations around the Earth (Methods). The amounts of organic matter in rock were classified into four bins on a global map of the Earth (Fig. 4). The low-hydrocarbon areas were further divided into oceanic crusts and continental crusts, resulting in six bins including the K–Pg case (Table 1). Areas containing high concentrations of organic matter were distributed mainly in and around continents, occupying narrow areas (Fig. 4). Areas of low concentration covered most of the Earth, especially in oceans. The amounts of surviving stratospheric soot after the impact were estimated to be 2–59 Tg BC in the low-hydrocarbon areas (occupying 68% of the Earth’s surface), 59–230 Tg BC in medium-hydrocarbon areas (occupying 20%), 230–590 Tg BC in high-hydrocarbon areas (occupying 10%), and 590–2300 Tg BC in very high-hydrocarbon areas (occupying 2%) (Methods; Tables 1 and 3; Fig. 4). This variation caused diverse climate changes depending on the impact site.

**Table 1:**
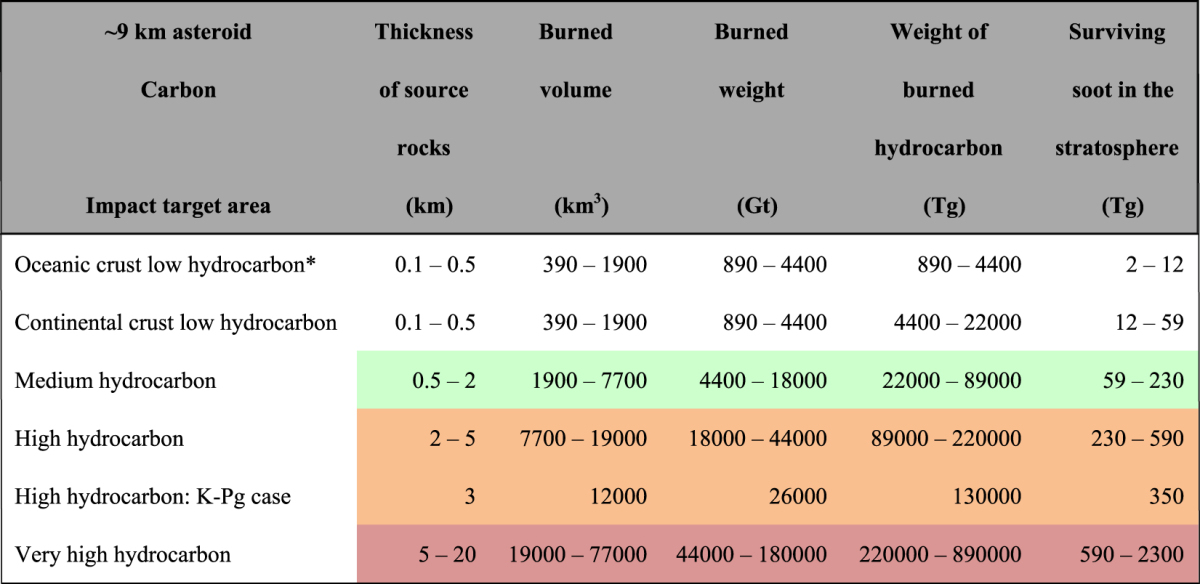
Amount of stratospheric soot produced by the impact of an asteroid at the K–Pg boundary on various target areas.

Burned volume: the product of source rock thickness^43^ and burned area. Burned weight: the product of burned area (35 × 35 × 3.14 = 3,850 km^2^ ^13,44^) and sedimentary rock density = 2.3 g/cm^3^. Surviving soot in the stratosphere refers to globally distributed soot after the impact (see Methods). Colors correspond to those in Fig. 4. Thickness of the K-Pg case, 3 km, is after Koeberl^54^ and Schulte *et al*.^5^. *Hydrocarbon/rock (TOC) is 0.1%. The other TOC are 0.5%.

**Table 2:**
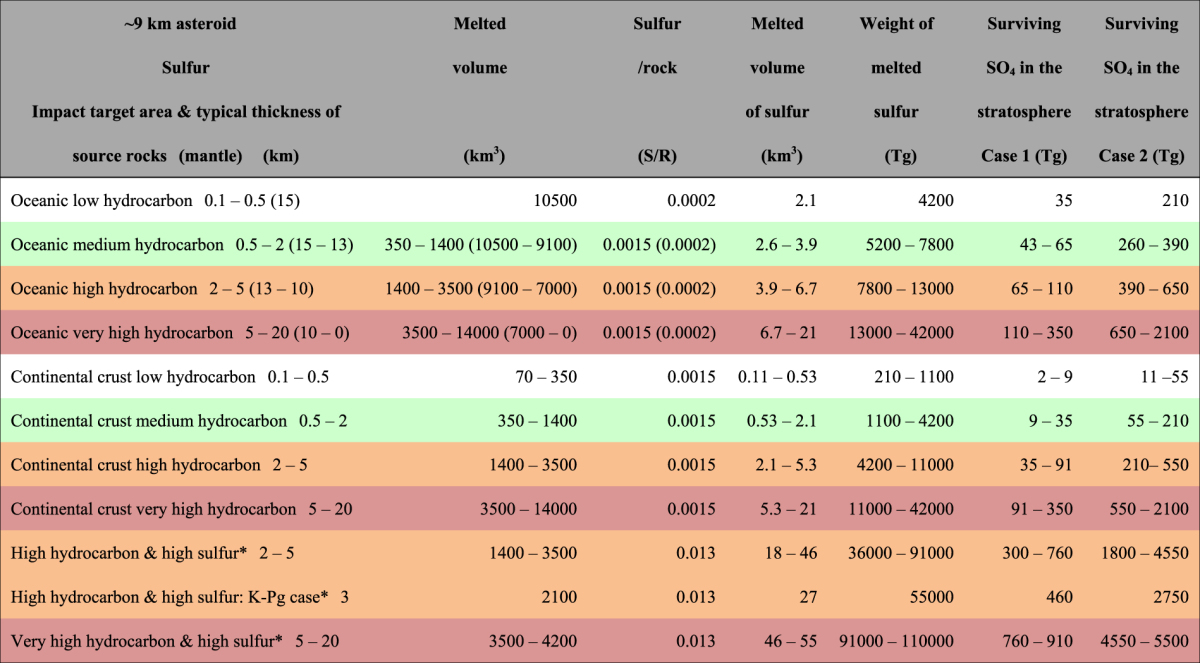
Amount of stratospheric sulfate produced by asteroid impact at the K–Pg boundary on various target areas.

Melted volume: the product of source rock thickness and melted area. Melted area (km^2^): 15 × 15 × 3.14 = 700 in the case of 45° oblique impact^44^. Sulfur/rock 0.013: 32/154 × 0.25 × 0.25 = 0.013 (154: the average of anhydrite and gypsum masses, 0.25: rate of thickness of evaporites^54^, 0.25: rate of sulfur in the breccia from a proximal ejecta deposit and evaporites, except for oxygen in Cretaceous sedimentary rocks near the crater^54^). Sulfur/rock 0.0015: product of 2400 ppm S in shale and 0.64 (rate of shales in sedimentary rocks^37^)^51^. Sulfur/rock 0.0002: 150–250 ppm S in the mantle^50,51^. (Sulfur in oceanic crust was not used due to its thinness, mostly 0.1–0.2 km.) Melted weight of sulfur is based on 2 g/cm^3^ density. Sedimentary rocks in approximately >6 km thick are mainly composed of mudstones and sandstones. Therefore, the amounts of sulfate in high sulfur and very thick sedimentary rocks are calculated for sedimentary rocks 5–6 km thick. See Methods for the process used to estimate the amount of surviving stratospheric sulfate (SO_4_). *continental crust.

The amounts of stratospheric sulfate that survived after the impact were calculated for 11 areas that included: i) the oceanic crust and mantle (occupying 68% of the Earth’s surface), ii) normal sulfur content areas on the continental crust (occupying 31% of the Earth’s surface), and iii) sulfate evaporite (anhydrite and gypsum)-rich (high-sulfur) areas on the continental crust (occupying 1% of the Earth’s surface [Supplemental Table 1]; approximately 15% of the orange and magenta areas on the continental crust (6.5%) in Fig. 4 correspond to sulfate evaporite-rich areas^22^). In case 1 (see Methods), based on Ohno *et al*.^12^, which considers the complete scavenging of sulfate produced from SO_3_ by large falling silicate particles just after the impact, this results in 35–350 Tg SO_4_, 2–350 Tg SO_4_, and 300–910 Tg SO_4_ for i), ii), and iii), respectively (Table 2). Approximately 6 times more sulfate survives in case 2, which assumes that all sulfur was ejected as sulfate and a survival rate of sulfate was the same as that of soot (Methods; Table 2).

### Climate change and mass extinction

**Figure 5:**
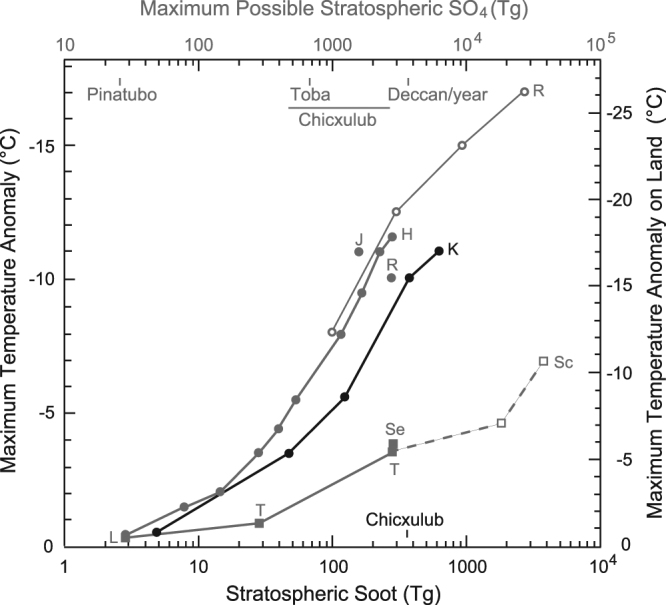
Relationships between the maximum global mean surface air temperature anomaly and globally distributed amounts of soot from asteroid impacts (black) and the maximum possible sulfate from volcanic eruptions (gray) injected into the stratosphere. The stratospheric soot amount and maximum temperature anomaly (left axis) are taken from Fig. 1a,c. The maximum temperature anomaly on land (right axis) is shown only for the stratospheric soot and is taken from Fig. 1a,d. The K^1^ curve was used for estimating the temperature anomalies for the amount of soot (Table 3). Relationships for sulfate and maximum temperature anomaly (left axis) are taken from published data^23–30^ calculated by various global models in volcanic eruption studies and are classified into three categories. The first category is taken from Robock *et al*.^23^ (open circles, marked by R). The second category is taken from Timmreck *et al*.^24,25^, Segschneider *et al*.^26^, and Laakso *et al*.^27^ (filled squares, marked as T, Se, L). Schmidt *et al*.^28^ (open squares, marked as Sc) belongs to this category, but this case gives 10 years of continuous injection of sulfur into the upper troposphere instead of a short-term injection to the stratosphere. The third category is taken from Robock *et al*.^23^, Jones *et al*.^29^, and Harris and Highwood^30^ (filled circles, marked as R, J, H). The HR^23,30^ curve (the first and third categories) and the LTS^24–28^ curve (the second category) were used to estimate the temperature anomalies for the sulfate amounts in the upper and lower cases, respectively (Supplemental Table 1). See the text for details. Amounts of stratospheric soot and sulfate ejected by the Chicxulub impact and sulfate by volcanic eruptions^23,25,27,28^ are also shown (Tables 1 and 2).

The level of climate change required to cause a mass extinction is assumed to be an approximately 8–10 °C decrease in global mean surface air temperature based on Kaiho *et al*.^1^. Previous soot modeling results^1^ and the current study provide information on relationships between the amount of globally distributed stratospheric soot ejected by the impact and the maximum global mean surface air temperature anomaly caused by the soot injection (Fig. 5). We estimated the climate changes (the maximum anomalies of global averaged values) caused by stratospheric soot ejected by asteroid impacts for various target areas using Fig. 5 (Table 3, Methods). When the asteroid hit high-hydrocarbon areas occupying 10.4% of the Earth’s surface (orange areas in Fig. 4; Tables 1 and 3), it resulted in an 8–11 °C decrease in global mean surface air temperature and a 13–17 °C decrease in global mean surface air temperature on land, these conditions resulted in mass extinction^1^.

**Table 3:**
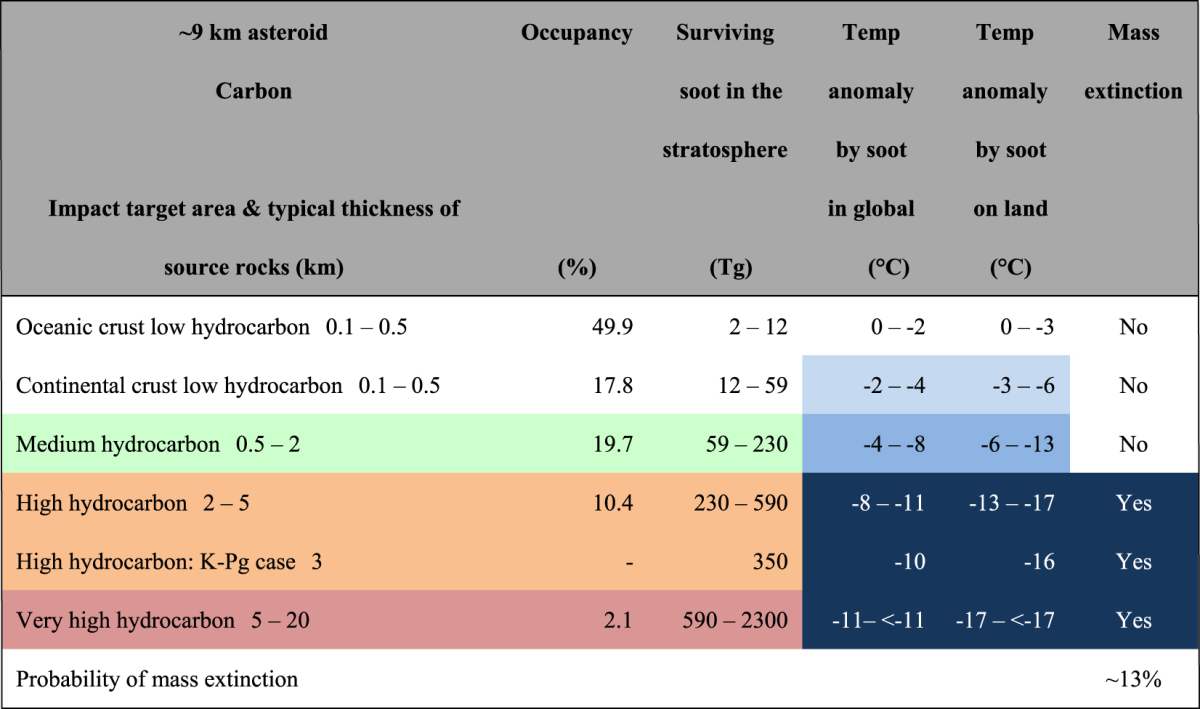
Summary of maximum global mean surface air temperature anomalies due to soot, the presence or absence of mass extinctions in various target areas, and the occupancy of each area.

Colors in the soot column correspond to those in Table 1. Color gradient from pale to deep blue indicates the scale of the global mean surface air temperature anomaly: the deepest blue areas correspond to surface air temperature anomaly causing a mass extinction. Temperatures were derived using the K curve in Fig. 5. Temp: surface air temperature.

When the asteroid hit very high-hydrocarbon areas, occupying 2.1% of the Earth’s surface (magenta areas in Fig. 4; Tables 1 and 3), it caused more severe cooling (i.e., ≥11 °C global cooling and ≥17 °C cooling on land), and resulted in mass extinction.

When the asteroid hit medium-hydrocarbon areas, occupying 19.7% of the Earth’s surface, respectively (olive areas in Fig. 4; Tables 1 and 3), 4–8 °C global cooling and 6–13 °C cooling on land. These climate changes corresponded to the case of no mass extinction given by Kaiho *et al*.^1^.

When the asteroid hit low-hydrocarbon areas on the continental crust occupying 17.8% of the Earth’s surface and on the oceanic crust occupying 49.9% (white areas in Fig. 4; Tables 1 and 3), this led to 2–4 °C and 0–2 °C global cooling and 3–6 °C and 0–3 °C cooling on land, respectively.

In addition to BC, stratospheric sulfate may also contribute to surface cooling. Figure 5 shows the relationships between the maximum possible stratospheric sulfate and the maximum global mean surface air temperature anomaly caused by the sulfate injection, prepared using published data^23–30^ calculated by various global models in volcanic eruption studies (Methods). It is difficult to quantitatively estimate climate changes caused by sulfate aerosols ejected by the impact using the results of volcanic eruption studies. Nevertheless, we estimated the surface air temperature anomaly potentially caused by the stratospheric sulfate using the sulfate amounts from 11 impact areas (Table 2) and the relationship shown in Fig. 5 (Methods) to identify the occurrence of a mass extinction caused only by sulfate. In case 1, most areas contained normal sulfate amounts (2–350 Tg surviving SO_4_ in the stratosphere), resulting in 0–4 °C global cooling due only to sulfate. Evaporite-rich areas have more sulfate (300–910 Tg surviving SO_4_ in the stratosphere), resulting in 1–7 °C global cooling due only to sulfate (Supplemental Table 1). These results suggest that sulfate has a limited contribution to cooling and that sulfate alone cannot trigger mass extinction. In case 2, 0–11 °C cooling in normal sulfate areas and 3–14 °C cooling in high sulfate areas could have occurred (Supplemental Table 1). Mass extinction may have occurred by only sulfate in case 2 when an asteroid hit high sulfate areas and very thick normal sulfate areas occupying approximately 1–2% of the Earth’s surface^22^, because sedimentary rocks are rarely more than 10 km thick (Supplemental Table 1).

These results suggest that climate changes (in terms of extinction levels) can be estimated using stratospheric soot amounts. Soot from hydrocarbon-rich areas (approximately 13%) including high sulfate areas limited to 1% of the Earth’s surface^22^ caused 8– ≥ 11 °C global cooling, 13– ≥ 17 °C cooling on land, a decrease in precipitation by approximately 70– ≥ 85% on land, a decrease of approximately 5– ≥ 7 °C in seawater temperature at a 50-m water depth, and mass extinction marked by extinction of dinosaurs^1^ (Figs 1 and 2; Table 3). At the time, these hydrocarbon-rich areas were marine coastal margins, where the productivity of marine algae was generally high and sedimentary rocks were thickly deposited (Fig. 4). Therefore, these areas contain a high amount of organic matter, part of which becomes soot with the heat of an impact. The Chicxulub impact occurred in a hydrocarbon-rich, sulfate-dominated area, and is a rare case of mass extinction being caused at such an impact site (Table 3 and Supplemental Table 1). Moreover, sulfate-rich areas overlapped with hydrocarbon-rich areas, so an impact in such a region would have caused concurrent ejection of sulfate and large amounts of soot, causing a mass extinction. Therefore, soot is likely to have been more important than sulfate as a cause of mass extinction induced by a bolide impact.

### Probability of mass extinction

**Figure 6:**
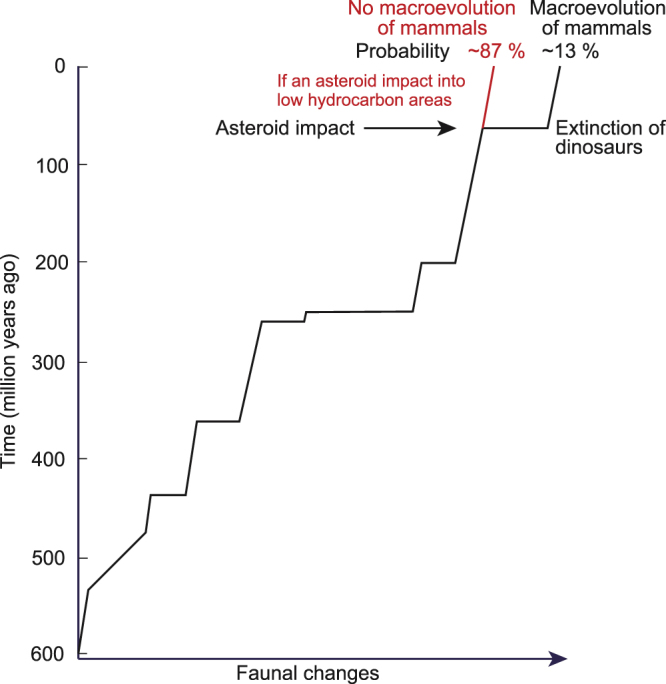
Phanerozoic faunal changes with approximately 13% probability following the Chicxulub asteroid impact. Changes in fauna are based on extinction rates. Changes through the K–Pg boundary mass extinction are enhanced by a change in the main terrestrial fauna from dinosaurs to mammals.

The probability of mass extinction at the K–Pg boundary was approximately 13% (10.4% [orange areas in Fig. 4; Table 3] plus 2.1% [magenta areas in Fig. 4; Table 3]), after the asteroid impacted Earth. The collapse of ecosystems with dinosaurs on land and large marine reptiles and ammonites in the sea at the top of the food chain^1^ was probably due to soot with possible contributions by sulfate from the Chicxulub asteroid impact^1,31^, and led to the subsequent macroevolution and diversification of mammals. Therefore, the low probability of mass extinction indicates the low probability of the subsequent macroevolution of mammals (Fig. 6).

### Summary

The probability of mass extinction occurring after an asteroid that could hit a random location on the Earth’s surface was approximately 13% when the Chicxulub-scale asteroid hit the Earth. Soot could be the main cause of mass extinction after an asteroid impact. The history of life on Earth could have varied, then, according to impact site, and depended on minute differences in the orbital forcing of asteroids. The probability of mass extinction was quite low even with an asteroid as large as the K/Pg bolide, because hydrocarbon-rich and sulfate-rich sites were rare. If the asteroid had hit a low–medium hydrocarbon area on Earth, mass extinction could not have occurred and the Mesozoic biota could have persisted beyond the K/Pg boundary.

## Methods

### Model calculation

We used a coupled atmosphere–ocean global climate model developed at the Meteorological Research Institute, MRI-CGCM3^32^. Detailed descriptions and evaluations of the model calculations are provided in the Supplementary Information of Kaiho *et al*.^1^. Following this method, we performed two 10-year experiments with the BC ejection due to the Chicxulub asteroid impact (using the 20-Tg and 200-Tg BC cases), based on pre-industrial climate conditions. We also used model calculation results for the 500-Tg, 1500-Tg, and 2600-Tg BC cases and a 30-year control experiment with no BC ejection^1^. In these cases, BC was ejected into one column of the model grid box at 21°N, 90°W (Yucatan Peninsula) in the current geographical setting using the vertical distribution obtained by Saito *et al*.^33^. We also performed an additional 10-year experiment for the high-latitude 200-Tg BC ejection case, where BC was ejected at the late-Eocene Popigai crater (71°N, 111°E); all other conditions were the same as those for the Chicxulub cases. In these calculations, we assumed spherical particles for atmospheric aerosols and their optical properties in the solar and terrestrial spectral range were calculated on the basis of microphysical data such as the size distribution and spectra refractive index using the software package Optical Properties of Aerosols and Clouds (OPAC)^34^. We evaluated the climate response due to BC ejection by subtracting the monthly climatology (30-year mean) of the control experiment from the monthly mean results of the other experiments.

The particle size distributions could affect the atmospheric lifetime of BC and its radiative effects. Evaluations of the climate effects caused by the size distributions of BC particles for the Chicxulub case are provided in the Supplementary Information of Kaiho *et al*.^1^. They conducted a 10-year sensitivity experiment for the 1500-Tg BC injection with a larger BC size distribution (i.e., mode radius of 43.7 nm and geometric standard deviation of 1.64 for the lognormal number size distribution), which was observed by recent aircraft measurements, and values were larger than the OPAC values (i.e., the mode radius of 11.8 nm and the geometric standard deviation of 2.00). Compared with the 1500-Tg BC case, this experiment for a larger BC size distribution led to less BC loading in the atmosphere, resulting in a smaller cooling effect amplitude (up to 1.5 °C difference in the monthly global mean surface air temperature; Supplemental Fig. 1). The maximum temperature difference due to the size distributions will be less than 1.5 °C for <500 Tg BC cases, because the amplitudes of the cooling effect of <500-Tg BC cases were smaller than those of the 1500-Tg BC case (Fig. 1). These results indicate that the temperature difference (<1.5 °C) caused by the different size distributions will be less than the maximum temperature anomalies caused by the different amounts of BC (i.e., 3 °C, 6 °C, and 10 °C for the 200, 500, and 1500-Tg BC cases, respectively). The climate model calculation assumed spherical particles for all aerosol species, although soot particles generally consist of aggregated carbon spherules. Numerical studies have shown that the aerosol optical properties (e.g., absorption) at visible wavelengths were enhanced by aggregation by no more than about 30%^35^. They have also shown that the relative difference in direct radiative forcing of soot particles between uncoated spheres and these aggregates was about 3% (global annual mean at the top of the atmosphere) in the present-day atmosphere^36^. These results suggest that climate changes caused by BC injection would be more greatly influenced by the amount of BC than the particle size and shape for the Chicxulub-scale asteroid impact.

### Hydrocarbon (organic carbon) content

The average weight of hydrocarbons in sedimentary rocks is 0.5%, based on the average organic carbon content (%) of shales, carbonates, and sandstones and their relative proportions^37^, with lower content in pelagic oceans (Table 1)^38^. The organic carbon content (%) in continental, coastal, and upwelling areas at the end of the Cretaceous was estimated at an average of 0.5 wt% and that in the pelagic oceans was estimated at an average of 0.1 wt%, based on the organic carbon content (%) of surface marine sediments^38^ and pre-Cenozoic sedimentary rocks^39–42^.

### Thickness of sedimentary rocks

The total thickness of sedimentary rocks in the present-day crust^43^ was revised by removing thick Cenozoic sedimentary rocks. Exceptionally thick Cenozoic sediments, such as those found in India due to the Himalayas, were removed and the white and olive areas were added in paleoceans located between the North and South American continents and between Asia and Africa–India at the end of the Cretaceous (Fig. 4). We also used the thickness ratio between the pre-Cenozoic and Cenozoic, or the thickness of the Cenozoic, in selected hydrocarbon-rich areas (orange and magenta areas, Fig. 4; Supplemental Table 2), to obtain the distribution of the total thickness of sedimentary rocks at the end of the Cretaceous. We divided the globe into four types of areas based on the thickness of sedimentary rocks: low-hydrocarbon areas (<0.5 km thick, mostly 0.1–0.2 km; approximately pelagic), medium-hydrocarbon areas (0.5–2 km thick; approximately hemipelagic), high-hydrocarbon areas (2–5 km thick), and very high-hydrocarbon areas (5–20 km thick); we then divided these types into oceanic and continental (composed of continental and shelf rocks) crusts, resulting in 12 bins. The areas (%) of the 12 regions were calculated using ArcGIS10.3 (ESRI, Redlands, CA, USA; Supplemental Table 3). All values are approximate; estimated values are sufficient to obtain approximate areas and the probabilities of mass extinctions.

### Amount of stratospheric soot

Impact velocities were constant in this calculation, because we assumed that the asteroid impact would be the same at random locations on the Earth’s surface. Temperature in the bulk impact-induced vapor is similar between 30° and 90° impact angles, but pressure depends strongly on the impact angles (higher angles correspond to higher pressure) in 10-km asteroid impact cases^44^. In the impact angle range, pressure rapidly decreases from > ~30 GPa to <~20 GPa at 5–10 km distance from the impact center on rocks^44^. An experimental study shows that soot-like materials were formed at 20–30 GPa and effect of pressure for soot formation is small at <20 GPa^45–47^. Therefore, all the impact-angle cases cause a change from soot-formation states to no soot-formation states at the 5–10 km distance. The volume of sedimentary rocks ejected into the stratosphere is similar in 15–90° impact angle cases (the maximum volume [30° impact angle for calcite] is 1.7 times of minimum volume [15° impact angle for calcite])^44^. There were no significant latitudinal differences in rates among organic carbon-rich areas (Supplemental Table 3). The volume of granite (crust) and mantle melt was higher at higher-impact angles, but granite and mantle rock are not a source of soot^33,44^. Overall, impact angles >30° did not likely to change significantly the probability of mass extinction.

An asteroid approximately 9 km in diameter ejects target rocks, including sedimentary rocks, crust, and mantle (in the case of oceanic crust impact), within a transient crater, i.e., the hole made during the initial impact, which has an estimated diameter of 80–110 km (the final crater size is 170 km)^48^. Temperatures inside this transient crater would have reached near the ignition point of hydrocarbon compounds (~600 K) within a diameter of about 70 km^13,44^. Therefore, all sedimentary rocks (usually < 5 km in thickness) and part of the continental crust would have been ejected in >15° oblique impacts on continents, and all sedimentary rocks (usually <0.5 km in thickness), oceanic crust (~5 km in thickness), and part of the mantle would have been ejected in >15° oblique impacts on oceans^44^. There are few hydrocarbons in the crust and mantle; therefore, we used only sedimentary rocks to estimate the amount of hydrocarbon. The product of the burned weight and averaged hydrocarbon content provides the amount of hydrocarbon ejected by the impact of a 9-km asteroid on the Earth (Table 1). The amount of stratospheric soot generally depends on the soot emission factor, the fraction of soot injection to the stratosphere (0.23 in the 90° impact angle case)^1^, and the surviving soot fraction remaining in the stratosphere due to the short-term rapid removal after the impact (e.g., sedimentation due to coagulation with large particles ejected by the impact). The efficiency of soot formation from hydrocarbon may be also dependent on the chemical composition and the redox conditions in the bulk impact-induced vapor. These factors are included in the soot emission factor (ranging 3–10%)^13,49^ and we used the average value (6.5%) in this study. The overall surviving fraction in ejected soot that could be spread globally in the stratosphere is assumed to be 4.2%, to fit the 1500-Tg BC K–Pg case (350 Tg BC in the global stratosphere), which may explain the extinction of dinosaurs and ammonites and the survival of crocodiles^1^. The surviving fraction of the 350-Tg BC case was applied to all BC cases and the amounts of surviving stratospheric soot after the impact were estimated for every case (Table 1). The effect of stratospheric soot on the global mean surface air temperature anomaly was estimated using the K curve shown in Fig. 5. The temperature reductions might be underestimated for  <350-Tg BC cases and overestimated for >350-Tg BC cases, because the surviving fraction of BC would decrease among the cases with more BC, probably due to the greater likelihood of particle-size growth by coagulation.

### Amount of stratospheric sulfate

The main source of sulfate aerosols is evaporites [anhydrite (CaSO_4_) and gypsum (CaSO_4_ 2H_2_O)] in sedimentary rocks deposited in a closed shallow sea on continental crust (the rate of sulfur/rocks is calculated as 13,000 ppm S in high-concentration areas, Table 2). The amount of sulfur from other sedimentary rocks is minor compared to that from evaporite-rich sedimentary rocks, so sulfur content was calculated as the average sulfur content in sedimentary rocks: 1500 ppm S (Table 2). The main source of sulfate aerosols in an oceanic crust impact is the mantle beneath the oceanic crust, because the volume of mantle materials ejected following an approximately 9-km asteroid impact is very large compared to oceanic crust and sedimentary rocks, calculated as having a 30-km diameter of melting^44^, 15-km thickness^44^, and 150–250 ppm sulfur content^50,51^ (Table 2). Recent impact experiments have shown that an impact produces a high sulfur trioxide (SO_3_)/sulfur dioxide (SO_2_) ratio, approximately 30 for asteroids and Jupiter family comets (3% SO_2_), whose velocity is approximately 20 km/s^12^. According to the results of those studies, SO_3_ cannot form global stratospheric sulfate aerosols because of the rapid formation of sulfuric acid aerosols, resulting in efficient scavenging of sulfuric acid aerosols (1 μm in size) due to coagulation with larger falling silicate dust particles (100 μm in size)^12^. These phenomena would occur near the impact location within a few days. Remaining SO_2_ in the stratosphere, corresponding to 3% of ejected sulfur, would gradually be converted into sulfate aerosols and would be spread globally (Table 2). We used two cases to estimate the amount of stratospheric sulfate (SO_4_) that survived after an impact. Following Ohno *et al*.^12^, the amount of surviving SO_4_ in case 1 was estimated to be the product of the weights of melted sulfur, the sulfur emission factor (0.4: (2000-1200)/2000 [ppm])^52^, the sulfur surviving fraction in the stratosphere due to short-term rapid removal after impact (0.03)^12^, the fraction of injection to the stratosphere (0.23), and the mass ratio of SO_4_/S (3), as summarized in Table 2. Case 2 assumed that all sulfur was ejected as sulfate and that the surviving rate of sulfate was the same as that of soot. The amount of sulfate surviving in the stratosphere in case 2 was estimated to be the product of the weights of melted sulfur, the sulfur emission factor (0.4), the surviving rate (0.042), and the mass ratio of SO_4_/S (3).

### Reason of survival of soot

In case 1 we assumed the removal of all SO_4_ produced from SO_3_ from the atmosphere after the impact and the survival of a portion of soot. SO_3_ and SO_2_ are released from rocks evaporated or melted by an impact that covers a 30-km-diameter area. Most silicate particles are sourced from silicate vapor from within the same impact area^11^. In contrast, soot is mainly formed in 70-km-diameter area. The difference of source areas could cause that the sulfate particles were efficiently scavenged from the atmosphere by large falling silicate particles in their path; however, soot particles were less scavenged resulting in their survival.

### Temperature anomaly caused by sulfate aerosols

We used published data^23–30^ calculated by various global models in volcanic eruption studies to estimate possible temperature anomalies caused by sulfate aerosols. The calculation methods and treatments of the sulfate aerosols in the models (e.g., injection height, location, time, gas-phase chemistry, aerosol microphysics, size distributions, and optical properties) differ between the models^23–30^, causing large variation in the resulting relationships shown in Fig. 5. The models can be roughly classified into three categories. The models in the first category calculate climate effects explicitly by treating gaseous SO_2_ concentration as volcanic emission, but do not treat aerosol size growth due to aerosol microphysics (e.g., coagulation), leading to large temperature anomalies likely due to the longer residence time of sulfate^23^ (Fig. 5). The models in the second category calculate climate effects by giving the aerosol optical depth, which is calculated by an another (offline) model that treats the effects of aerosol growth and sedimentation more realistically, resulting in smaller temperature anomalies that are likely due to shorter residence time^24–28^. The models in the third category calculate climate effects using sulfate loadings derived from the aerosol optical depth dataset, which yields temperature anomalies between those of the first and second categories (but with results closer to those of the first category models)^23,29,30^. We simply converted the initially given SO_2_ mass in the stratosphere to the stratospheric SO_4_ mass using molecular weights for some of the model results in Fig. 5, although the maximum SO_4_ loading would be smaller than the initial SO_2_ amount, because the peak SO_4_ loading would appear a few years after the initial SO_2_ injection. Therefore, we referred to the stratospheric SO_4_ as the maximum possible stratospheric SO_4_ in Fig. 5. Considering the high variation in the relationship shown in Fig. 5, we estimated the maximum global mean surface air temperature anomaly using the HR^23,30^ curve (upper case, the first and third categories) and LTS^24–28^ curve (lower case, the second category) for the stratospheric SO_4_ in every impact scenario in Table 2 for cases 1 and 2 (Fig. 5; Supplemental Table 1).

## Electronic supplementary material

Supplementary information
