# Supplementary material for: Site of asteroid impact changed the history of life on Earth: the low probability of mass extinction
Source: Sci Rep. 2017 Nov 9;7:14855. doi: 10.1038/s41598-017-14199-x (PMC5680197; doi:10.1038/s41598-017-14199-x)
Supplement: Supplementary file 1 — Supplementary information [file 41598_2017_14199_MOESM1_ESM.pdf]

Site of asteroid impact changed the history of life on Earth: the low probability of mass extinction: Supplementary information

Kunio Kaiho[1] & Naga Oshima[2]

[1]Department of Earth Science, Graduate School of Science, Tohoku University, Sendai 980-8578, Japan. [2]Meteorological Research Institute, Tsukuba 305-0052, Japan. Correspondence and requests for materials should be addressed to K.K. (email: Kaiho@m.tohoku.ac.jp)

We referred to the literature[55–97] in Supplemental Table 2 in removing the Cenozoic sedimentary rock thickness data from the total calculated thickness of the sedimentary rocks of the continental crust in areas of high hydrocarbon content (orange and magenta areas in Figure 4).

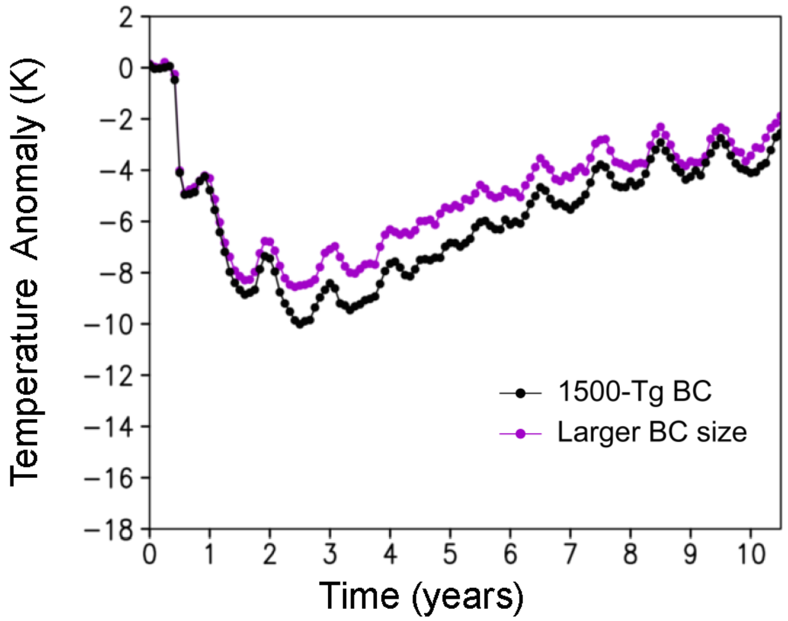

Supplemental Figure 1: Model sensitivity experiments for the surface air temperature changes for the 1500-Tg BC Chicxulub case. Changes in the global averages of surface air temperature for the 1500-Tg BC case (black) and the experiment with a larger BC particle size distribution (purple) calculated by the climate model. Monthly anomalies from the control experiment (no ejection case) are shown. See the text (Methods) for details.

Supplemental Table 1: Summary of maximum global mean surface air temperature anomalies due to soot and sulfate, presence or absence of mass extinctions in various target areas, and occupancy of each area

~9 km asteroid Carbon and sulfur Impact target area & typical thickness of source rocks (mantle) (km)	Occupancy (%)	Surviving soot in the stratosphere (Tg)	Temp anomaly by soot in global (°C)	Temp anomaly by soot on land (°C)	Surviving SO_4 in the stratosphere Case 1 (Tg)	Surviving SO_4 in the stratosphere Case 2 (Tg)	Temp anomaly by SO_4 in global Case 1 (°C)	Temp anomaly by SO_4 in global Case 2 (°C)	Mass extinction
Oceanic low hydrocarbon 0.1 – 0.5 (15)	49.9	2 – 12	0 – -2	0 – -3	35	210	-0.5	-1 – -3	No
Oceanic medium hydrocarbon 0.5 – 2 (15 – 10)	12.4	59 – 230	-4 – -8	-6 – -13	43 – 65	260 – 390	-0.5 – -1.5	-1 – -4	No
Oceanic high hydrocarbon 2 – 5 (13 – 10)	5.2	230 – 590	-8 – -11	-13 – -17	65 – 110	390 – 650	-0.5 – -2	-1 – -6	Yes
Oceanic very high hydrocarbon 5 – 20 (10 – 0)	0.8	590 – 2300	-11– <-11	-17 – <-17	110 – 350	650 – 2100	-0.5 – -4	-2 – -11	Yes
Continental crust low hydrocarbon 0.1 – 0.5	17.8	12 – 59	-2 – -4	-3 – -6	2 – 9	11 –55	0	0 – -0.5	No
Continental crust medium hydrocarbon 0.5 – 2	7.3	59 – 230	-4 – -8	-6 – -13	9 – 35	55 – 210	0 – -1	0.5 – -3	No
Continental crust high hydrocarbon 2 – 5	4.4	230 – 590	-8 – -11	-13 – -17	35 – 91	210– 550	-0.5 – -2	-1 – -6	Yes
Continental crust very high hydrocarbon 5 – 20	1.1	590 – 2300	-11– <-11	-17 – <-17	91 – 350	550 – 2100	-0.5 – -4	-2 – -11	Yes
High hydrocarbon & high sulfur* 2 – 5	0.8	230 – 590	-8 – -11	-13 – -17	300 – 760	1800 – 4550	-1 – -7	-3 – -13	Yes
High hydrocarbon & high sulfur: K-Pg case* 3	-	350	-10	-16	460	2750	-1 – -5	-3 – -11	Yes
Very high hydrocarbon & high sulfur* 5 – 20	0.2	590 – 2300	-11– <-11	-17 – <-17	760 – 910	4550 – 5500	-2 – -7	-4 – -14	Yes
Probability of mass extinction									~13%

Colors in soot and SO_4 columns correspond to those in Tables 1 and 2. The color gradient from pale to deep blue indicates the scale of

global mean surface air temperature anomaly. The deepest blue areas correspond to surface air temperature anomaly causing a mass extinction. Temperatures were derived using the K curve for soot and HR (upper case) and LTS (lower case) curves for sulfate in Fig. 5. See the text for case 1 and case 2 details. *continental crust. Temp: surface air temperature.

Supplemental Table 2: Ratio between pre-Cenozoic sedimentary rock thickness and total thickness or thickness of the Cenozoic sediments to obtain pre-Cenozoic sedimentary rock thickness for orange and magenta areas in Fig. 4.

Area of high organic carbon content	Pre-Cenozoic/Total	Thickness of the Cenozoic (km)	Reference
Off eastern Africa		0.6	*55*
Off southwestern Africa	0.7	0.1-0.5	*56-58*
Congo	>0.9		*59*
Off northwestern Africa	0.3		*60*
Sahara	0.8		*61*
Mediterranean	0.5		*62, 63*
Northern Arabia	>0.9		*64*
Black Sea	0.8		*65, 66*
Caspian Sea	0.8-0.9		*67, 68*
Northern Europe	>0.9		*69*
Off Northern Europe		0.5	*70*
Europian Arctic	0.9		*71*
Western Siberia	>0.9		*72*
Central Siberia	>0.9		*73, 74*
Eastern Siberia	>0.9		*75*
Central China	>0.9		*74, 75*
Tarim Basin	>0.9		*76*

Turkmenistan	>0.9		*77*
Himalayas	>0.9		*74, 78*
Arabian Sea		>5	*79*
Bay of Bengal	0.2		*80*
Off Northwestern Australia	0.4, 0.8		*81, 82*
Australia basins	>0.9		*83*
Off Antarctica		1.0	*84*
Andes	>0.9		*85*
Off Southeastern South America	~0.6	0.4	*86*
Off Northern South America	0.6		*57*
Gulf Coast	0.9		*87*
Gulf of Mexico	0.9		*88*
Yucatan	0.9		*54*
Off Eastern North America		0.3-1.0	*89, 90*
Rocky Mountains	>0.9		*91*
Aleutian Basin		1.0	*92, 93*
Canadian Arctic	0.9		*94, 95*
Off western Greenland		~1	*96*
Off eastern Greenland		0.9	*97*

Supplemental Table 3: Area (km^2) of each region defined by latitude, geography, and amount of organic matter

Color in Figure 1	Area	N60-90	N30-60	N0-30	S0-30	S30-60	S60-90	Total	Occupancy (%)
White	Ocean	5,291,066	37,570,230	75,060,124	75,630,904	52,083,568	9,847,780	255,483,673	49.9
	Shelf	890,730	1,063,014	454,537	16,582	105,627	-	2,530,491	0.5
	Continent	5,111,671	24,284,360	16,487,641	23,832,231	10,496,692	8,184,455	88,397,051	17.3
White total		11,293,468	62,917,604	92,002,302	99,479,718	62,685,887	18,032,236	346,411,214	67.7
Olive	Ocean	6,591,556	5,305,688	12,490,795	12,542,679	14,807,865	11,456,461	63,195,044	12.4
	Shelf	468,236	1,657,506	283,731	109,059	2,007,997	109,826	4,636,355	0.9
	Continent	1,416,107	11,028,895	6,122,278	7,372,063	5,233,596	1,430,787	32,603,727	6.4
Olive total		8,475,899	17,992,090	18,896,803	20,023,801	22,049,459	12,997,074	100,435,127	19.6
Orange	Ocean	5,810,662	2,028,152	7,912,811	4,540,188	4,225,328	2,231,690	26,748,831	5.2
	Shelf	608,395	1,813,977	1,056,168	299,063	1,355,842	77,583	5,211,027	1
	Continent	5,020,133	6,334,041	4,620,573	2,682,041	2,281,019	599,636	21,537,444	4.2
Orange total		11,439,189	10,176,170	13,589,552	7,521,292	7,862,189	2,908,909	53,497,302	10.5
Magenta	Ocean	1,651,169	250,409	1,279,557	388,808	420,149	289,145	4,279,237	0.8
	Shelf	272,748	872,857	1,006,660	99,113	320,391	11,561	2,583,329	0.5
	Continent	1,159,586	1,575,847	1,036,784	303,108	237,951	-	4,313,275	0.8
Magenta total		3,083,503	2,699,113	3,323,000	791,028	978,491	300,706	11,175,841	2.2
Total		34,292,059	93,784,977	127,811,658	127,815,840	93,576,025	34,238,924	511,519,483	

References

55. Simpson, Z. S. W., Schlich, R. *et al. Proceedings of the Ocean Drilling Program, Scientific Results* **25** (1974).
56. Hayes, D. E., Pimm, A. C. *et al. Proceedings of the Ocean Drilling Program, Scientific Results* **14** (1972).
57. Bolli, H. M., Ryan, W. B. F. *et al. Proceedings of the Ocean Drilling Program, Scientific Results* **40** (1978).
58. Hay, W. W., Sibuet, J.-C. *et al. Initial Reports of the Deep Sea Drilling Project* **75** (1984).
59. Wendorff, M. Stratigraphy of the Fungurume Group - evolving foreland basin succession in the Lufilian fold-thrust belt, Neoproterozoic-Lower Palaeozoic, Democratic Republic of Congo. *South African Journal of Geology* **106,** 17–34 (2003).
60. Lancelot, Y. & Seibold, E., *et al. Initial Reports of the Deep Sea Drilling Project* **41** (1977).
61. Bertrand, J. M. L., Caby, R., Ducrot, J., Lancelot, J., Moussine-Pouchkine, A. & Saadallah, A. Geology, structural development, U/Pb geochronology, tectonic implications for the hoggar shield. *Precambrian Research* **7,** 349–376 (1978).
62. Melki, F., Zouaghi, T., Chelbi, M. B. & Be´dir, M. Tectonic-sedimentary events and geodynamic evolution of the Mesozoic and Cenozoic basins of the Alpine Margin, Gulf of Tunis, north-eastern Tunisia offshore. *Comptes Rendus Geoscience* **342,** 741–753 (2010).
63. Bosellini, A. Dinosaurs ‘‘re-write’’ the geodynamics of the eastern Mediterranean and the paleogeography of the Apulia Platform. *Earth-Science Reviews* **59,** 211– 234 (2002).
64. Ibrahim, M. W. Petroleum geology of southern Iraq. *Bulletin-American Association of Petroleum Geologists* **67,** 97–130 (1983).
65. Robinson, A. G., Banks, C. J., Rutherford, M. M., Hirst, J. P. P. Stratigraphic and structural development of the Eastern Pontides, Turkey. *Journal of the Geological Society, London* **152,** 861–872 (1995).
66. Tüysüz, O., Yilmaz, İ. Ö., Švábenická, L. & Kirici, S. *The Unaz Formation:* A Key Unit in the Western Black Sea Region, N Turkey. *Turkish Journal of Earth Sciences* **21,** 1009–1028 (2012).
67. Rabinowitz, P. D., Yusifov , M. Z., Arnoldi, J. & Hakim, E. Geology, Oil and Gas Potential, Pipelines, and the Geopolitics of the Caspian Sea Region. *Ocean Development & International Law* **35,** 19–40 (2004).

68. Robert, A. M. M. *et al.* Structural evolution of the Kopeh Dagh fold-and-thrust belt (NE Iran) and interactions with the South Caspian Sea Basin and Amu Darya Basin. *Marine and Petroleum Geology* **57,** 68–87 (2014).

69. Nielsen, A. T. & Schovsbo, N. H. The Lower Cambrian of Scandinavia: Depositional environment, sequence stratigraphy and palaeogeography. *Earth-Science Reviews* **107,** 207–310 (2011).

70. Talwani, M. & White, S. M. Introduction and explanatory notes, Leg 38, Deep Sea Drilling Project. *Proceedings of the Ocean Drilling Program, Scientific Results* **38,** 3-56 (1976).

71. Shipilov, E. V. Late Mesozoic Magmatism and Cenozoic Tectonic Deformations of the Barents Sea Continental Margin: Effect on Hydrocarbon Potential Distribution. *Geotectonics* **49,** 53–74 (2015).

72. Vyssotski, A. V., Vyssotski, V. N. & Nezhdanov, A. A. Evolution of the West Siberian Basin. *Marine and Petroleum Geology* **23,** 93–126 (2006).

73. Pisarevsky, S. A., Natapov, L. M., Donskaya, T. V., Gladkochub, D. P. & Vernikovsky, V. A. Proterozoic Siberia: A promontory of Rodinia. *Precambrian Research* **160,** 66–76 (2008).

74. Ren, J. *et al.* Advances in research of Asian geology—A summary of 1:5M International Geological Map of Asia project. *Journal of Asian Earth Sciences* **72,** 3–11 (2013).

75. Hsu, K. J., *et al.* Tectonics of South China; Key to understanding West Pacific geology. *Tectonophysics* **183,** 9–39 (1990).

76. Chen, Y., Xu, B., Zhan, S. & Li, Y. First mid-Neoproterozoic paleomagnetic results from the Tarim Basin (NW China) and their geodynamic implications. *Precambrian Research* **133,** 271–281 (2004).

77. Brookfield, M. E. & Hashmat, A. The geology and petroleum potential of the North Afghan platform and adjacent areas (northern Afghanistan, with parts of southern Turkmenistan, Uzbekistan and Tajikistan). *Earth-Science Reviews* **55,** 41–71 (2001).

78. Yin, A. *et al.* Geologic correlation of the Himalayan orogen and Indian craton: Part 2. Structural geology, geochronology, and tectonic evolution of the Eastern Himalaya. *GSA Bulletin* **122,** 360–395 (2010).

79. Clift, P. & Gaedicke, C. Accelerated mass flux to the Arabian Sea during the middle to late Miocene. *Geology* **30,** 207–210 (2002).

80. Curray, J. R., Emmel, F. J. & Moore, D. G. The Bengal Fan: morphology, geometry, stratigraphy, history and processes. *Marine and Petroleum Geology* **19,** 1191–1223 (2002).

81. Dumoulin, J. A. & Bown, P. P. Depositional history, nannofossil biostratigraphy, and correlation of Argo Abyssal Plain Sites 765 and 261. *Proceedings of the Ocean Drilling Program, Scientific Results* **123,** 3–56 (1992).

82. Exon, N. F. & Buffler, R. T. Mesozoic seismic stratigraphy and tectonic evolution of the western Exmouth Plateau. *Proceedings of the Ocean Drilling Program, Scientific Results* **122,** 61–81 (1992).

83. Hill, A. C. & Walter, M. R. Mid-Neoproterozic (~830–750 Ma) isotope stratigraphy of Australia and global correlation. *Precambrian Research* **100**, 181-211 (2000).

84. Hayes, D. E. & Frakes, L. A. General synthesis deep sea drilling project Leg 28. *Proceedings of the Ocean Drilling Program, Scientific Results* **28,** 919–942 (1973).

85. Drobe, J., Lindsay, D., Stein, H. & Gabites, J. Geology, Mineralization, and Geochronological Constraints of the Mirador Cu-Au Porphyry District, Southeast Ecuador. *Economic Geology,* **108,** 11 –35 (2013).

86. Supko, P. R., Perch-Nielsen, K *et al. Initial Reports of the Deep Sea Drilling Project* **39,** (1977).

87. Hanor, J. S. & Mcintosh, J. C. Diverse origins and timing of formation of basinal brines in the Gulf of Mexico sedimentary basin. *Geofluids* **7,** 227–237 (2007).

88. Wilhelm, O. & Ewing, M. Geology and history of the Gulf of Mexico. *Geological Society of America Bulletin* **83,** 575–600 (1972).

89. Watkins, D. K. *et al.* Paleontological results from Ocean Drilling Program Leg 101. *In* Austin, J. A., Jr. & Schlager, W. et al. *Proceedings of the Ocean Drilling Program, Scientific Results* **101,** 473-480 (1988).

90. McCracken, S. R., Compton, J. & Hicks, K. Sequence-stratigraphic significance of glaucony-rich lithofacies at site 903. *Proceedings of the Ocean Drilling Program, Scientific Results* **150,** 171–187 (1992).

91. Begin, N. J. & Spratt, D.A. Role of transverse faulting in along-strike termination of Limestone Mountain Culmination, Rocky Mountain thrust-and-fold belt, Alberta, Canada. *Journal of Structural Geology* **24,** 689–707 (2002).

92. Creager, J. S., Scholl, D. W. *et al. Proceedings of the Ocean Drilling Program, Scientific Results* **19** (1973).

93. Sukhov, A. N., Chekhovicha, V. D. , Landerb, A. V., Presnyakovc, S. L. & Lepekhina, E. N. Age of the Shirshov Submarine Ridge Basement (Bering Sea) Based on the Results of Investigation of Zircons Using the U–Pb SHRIMP Method. *Doklady Earth Sciences* **439,** 926– 932 (2011).

94. Grantz, A. *et al.* Phanerozoic stratigraphy of Northwind Ridge, magnetic anomalies in the Canada basin, and the geometry and timing of rifting in the Amerasia basin, Arctic Ocean. *GSA Bulletin* **110,** 801–820 (1998).

95. Hadlari, T., Midwinter, D., Galloway, J. M., Dewing, K. & Durbano, A. M. Mesozoic rift to post-rift tectonostratigraphy of the Sverdrup Basin, Canadian Arctic. *Marine and Petroleum Geology* **76,** 148–158 (2016).

96. Srivastata, S. P., Authur, M., Clenent, B. et al. *Proceedings of Initial Reports of the Ocean Drilling Program* **105** (1987).

97. Hull, D. M., Osterman, L. E. & Thiede, J. Biostratigraphic synthesis of Leg 151, North Atlantic-Arctic Gateways*. Proceedings of the Ocean Drilling Program, Scientific Results* **151,** 627–644 (1996).
